# An Integrative Path Model of Healthcare Utilization Determinants in Traditional Korean Medicine and Western Medicine Based on the Anderson Behavioral Model

**DOI:** 10.3390/healthcare13020182

**Published:** 2025-01-18

**Authors:** Minjung Park

**Affiliations:** Department of Public Health & Administration, Seoul Digital University, 424 Gonghangdaero, Gangseo-gu, Seoul 07654, Republic of Korea; mjimage@hanmail.net

**Keywords:** Andersen behavioral model, healthcare utilization, traditional medicine, structural equation modeling, Korean medicine

## Abstract

Background: This study aimed to elucidate the determinants of healthcare utilization in South Korea’s dual healthcare system, encompassing both Western medicine and Korean medicine, through the Andersen Model. An integrative non-recursive path model of healthcare utilization determinants was proposed and analyzed. Methods: A path analysis using structural equation modeling, followed by mediation analysis, was conducted to determine the magnitude of the effect for each pathway. The 2020 data from the Korea Health Panel were used. Results: Structural equation modeling revealed that need factors, such as chronic diseases, disability, and self-rated health status, were the most significant drivers of both WM and KM utilization, while enabling factors significantly impacted WM utilization. Interestingly, WM and KM utilization were found to positively influence each other, contradicting the assumption of substitutional healthcare use. Mediation analysis further highlighted the interconnected nature of healthcare utilization pathways, with enabling and need factors showing significant indirect effects on utilization via the counterpart system. These findings underscore the importance of considering mutual influences in healthcare policy and resource allocation to enhance accessibility and efficiency. While the cross-sectional nature of the data limits causal inference, this study offers a robust theoretical framework and practical insights for addressing the complexities of healthcare utilization in dual-system contexts. Conclusion: The findings reveal that when traditional medicine functions within the national healthcare system, both Western medicine and traditional medicine are influenced by similar determinants in the same environment. Future research should explore longitudinal data to confirm these findings and investigate potential moderating effects of predisposing and enabling factors on WM and KM interactions.

## 1. Introduction

The study of determinants of healthcare utilization can identify factors influencing healthcare utilization in populations, predict future healthcare trends, and help design and implement effective health policies to improve healthcare accessibility. It also contributes to the efficient and fair allocation of limited healthcare resources based on an understanding of vulnerable groups and the enhancement in the healthcare system. Since healthcare utilization is influenced by various individual and contextual factors, a reasonable starting point for analyzing healthcare utilization is defining a theoretical framework. There are several theories identifying predictors of healthcare utilization [1], among which one of the most comprehensive and widely used frameworks is the behavioral model developed by R. Andersen and J.F. Newman in 1973 [2]. This socio-behavioral model defines healthcare services as part of a socio-cultural complex structure, considering various factors such as sociopsychological factors, personal factors, environmental factors, and institutional factors in predicting healthcare service utilization and identifying related determinants. The Andersen Behavioral Model of healthcare utilization (hereinafter referred to as the Andersen Model) has not only demonstrated the validity of factor classification in many previous studies but also integrated internal and external factors within individuals, being accepted as an analytical framework for behavioral prediction [3].

South Korea has a unique dual healthcare system that encompasses both Western medicine (WM) and traditional Korean medicine (KM). In studying the determinants of healthcare utilization in South Korea, it is essential to consider both KM and WM for the following reasons: First, both medical systems play significant roles in the healthcare management of Koreans, with their concurrent use being common practice. Second, an integrative approach enables a more comprehensive and accurate understanding of healthcare utilization patterns and health outcomes. Third, such an approach is crucial for deriving complete and reliable research findings.

However, previous studies have focused on only one side of the healthcare system (either KM or WM) [4,5], or compared the determinants of two healthcare systems separately [6,7]. Some studies performed an analysis using two variables as an independent variable and a dependent variable through a simple regression model, which did not reflect the mutual endogeneity in causal relationships [8], thereby failing to construct a comprehensive model of healthcare utilization determinants.

In the United States, Fouladbakhsh, J. M. and M. Stommel [9] presented a new conceptual framework of complementary and alternative medicine (CAM), the CAM Healthcare Model, which modifies the Andersen Model. This model incorporates predisposing, enabling, and need factors that affect CAM utilization. Finally, it suggested as a future task the need for an integrated model that simultaneously considers CAM and conventional medicine, since the use of CAM is shown to potentially influence the utilization of conventional health services.

Regression analysis, commonly used in quantitative research, is a statistical method most commonly used in behavioral sciences to identify the relationship and assess the strength and direction between predictor (X) and response (Y) variables [10]. However, it has limitations in modeling complex realities because it is difficult to model indirect relationships or mediation effects between variables beyond the relationship between two variables (X and Y). Over the past few decades, the emergence of diverse data types, complex data structures, and advanced behavioral theories discussed in empirical research has sparked a diversification of research methodologies based on linear regression models [11].

To overcome these limitations of regression analysis, it is possible to enhance the quality of behavioral science theories by including a third variable, such as a mediator (M) or a moderator (W), in the linear relationship between the predictor variable and the response variable (X→Y). The mediator acts as a linking mechanism that explains the relationship between the predictor and the response. In terms of statistical methodology, structural equation modeling can clearly demonstrate the paths, verifying the step-by-step effects between variables. It examines whether X affects M, which in turn affects Y, thereby decomposing the total effect of X on Y into direct and indirect effects. The direct effect refers to the pathway from X to Y, while the indirect effect captures the influence of X on Y through M. It is widely applied in social, behavioral, and health sciences to uncover underlying relationships and provide insights into causality.

Therefore, this study aims to verify not only the comprehensive determinants of healthcare utilization but also the mutual effects between the two healthcare systems by constructing a non-recursive structural equation model that can analyze the determinants of both traditional and Western medicine utilization within a single model, considering their endogenous relationship. This study differs from existing studies in that it not only examines the mutual mediating effects of the utilization of traditional medicine and Western medicine, but also the predisposing, enabling, and need factors of the Andersen Model. It is expected that more meaningful discussions will take place by exploring mutual influences through the results.

## 2. Materials and Methods

### 2.1. Integrative Conceptual Model of Healthcare Utilization in WM and KM

The path of both WM and KM can also be understood through the lens of the Andersen Model, which has been applied in various studies to assess factors influencing the utilization of healthcare. According to the Andersen Model, predisposing factors are characteristics that individuals already possess regardless of their will before the need for healthcare arises, including demographic characteristics such as age and gender, as well as socioeconomic factors like education and social class. Enabling factors pertain to the means and abilities to utilize healthcare services, including economic and sociological factors such as income level and family resources, and the accessibility to healthcare services. Need factors are physiological and psychological factors related to an individual’s level of disability or disease, which are direct causes for utilizing healthcare services [2,12,13].

A review of papers on the determinants of healthcare utilization in WM and KM found that most studies investigated WM and KM determinants of healthcare utilization separately according to their interests. It was confirmed that most studies included similar common measurement variables in each area, although they did not present an integrated model (Table 1).

In this study, five latent variables such as the predisposing factor, enabling factor, need factor, KM healthcare utilization (KMHCU), and WM healthcare utilization (WMHCU) were set. As predictors of the Anderson model, predisposing factors consist of age, gender, marriage, education, and region. Enabling factors include Private Health Insurance coverage (PHI) and occupation as measurement variables. Need factors are measured by self-rated health status (SRH), the number of chronic diseases (CHRONIC), the presence of disability (DISABILITY), and the need for care (CARE), which is a condition in which a person requires assistance due to difficulties in performing daily activities, social interactions, or leisure activities as a result of illness, injury, or other health-related issues.

Among these variables, the most debated ones are region and occupation. Systematic reviews analyzing large-scale studies have shown that region and occupation are sometimes classified as predisposing factors and, at other times, as enabling factors. As a predisposing factor, region is understood as a comprehensive environmental element that influences individual healthcare utilization [26]. On the other hand, as an enabling factor, region can be interpreted as a proxy variable reflecting healthcare accessibility, thereby facilitating healthcare utilization [27]. Occupation can also be categorized as a predisposing factor in its traditional sense [2,26]; however, it can also be considered an enabling factor as it can reflect an individual’s economic activities and socioeconomic status [14,26,27]. In this study, region and occupation were initially assigned to each of the two categories, respectively, and pilot analyses were conducted repeatedly. Based on the results of analysis, region was ultimately classified as a predisposing factor and occupation was categorized as an enabling factor due to its higher model fit, and the main analysis was conducted with this classification.

**Figure 1:**
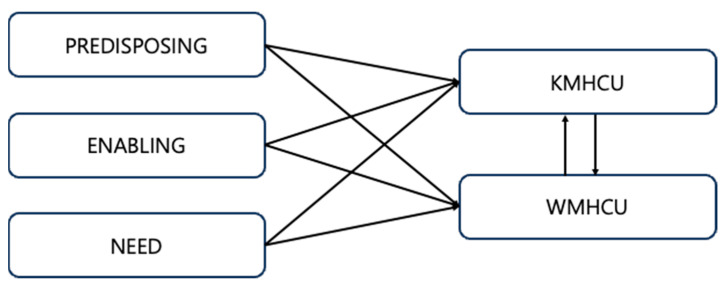
Integrative conceptual model of healthcare utilization in Western medicine and Korean medicine. KMHCU: Korean medicine healthcare utilization, WMHCU: Western medicine healthcare utilization.

Healthcare utilization as a response variable is divided into KMHCU and WMHCU. WMHCU is measured by the number of inpatient treatment days (DAYS OF INPATIENT CARE) and hospitalization costs (INPATIENT CARE COST), and the number of outpatient treatment days (DAYS OF OUTPATIENT CARE) and outpatient costs (OUTPATIENT CARE COST). Considering that KMHCU has a relatively small number of hospitalizations, it was measured by the number of outpatient treatment days (DAYS OF OUTPATIENT CARE) and outpatient costs (OUTPATIENT CARE COST) (Table 2). Finally, we conceptualized that each predictor affects KMHCU and WMHCU, and that KMHCU and WMHCU also influence each other (Figure 1).

### 2.2. Research Subjects

For this study, data from the 2020 wave of the Korea Health Panel, constructed by a consortium of the National Health Insurance Service and the Korea Institute for Health and Social Affairs, were used. The Korea Health Panel (KHP), which aims to improve the responsiveness, accessibility, and efficiency of healthcare services for the population, newly constructed its second wave data using the 2016 registered census as a sampling frame. A two-stage stratified cluster sampling method was used, considering the squared root proportional distribution and design effect for 17 cities and provinces. After the first stratification, a proportional distribution was applied by dividing urban and rural areas for the second stratification, resulting in samples from 708 sample survey districts targeting 8500 households and their members. The main survey content consists of four areas for households, six areas for individuals, and one area for both households and individuals. This two-stage stratified cluster sampling method used in the KHP is an efficient approach that ensures the representativeness of the overall population. By incorporating stratification that considers urban, rural, and regional characteristics, it better represents the national population.

Healthcare utilization among adolescents under the age of 19 is largely dependent on the decision making of parents or guardians, and they have limited autonomy in exercising their own healthcare choices. Therefore, this study focused on adults aged 19 and older to enhance the accuracy and validity of the analysis of the determinants of healthcare utilization.

### 2.3. Data Analysis

Descriptive statistical analysis was conducted to examine the demographic characteristics of the study subjects. Structural equation modeling was applied to explore the determinants of WM and KM utilization and their mutual mediation relationship according to the Andersen Model. The Bootstrapping technique was conducted for direct statistical verification of mediation effects. This test forms an empirical sampling distribution through numerous theoretical samples, and then verifies the coefficients through Bootstrap confidence intervals, offering valid effectiveness verification results even if the sampling distribution does not follow a normal distribution [28]. A total of 1000 samples were generated to calculate the 95% confidence interval for mediation effects, with all statistical processing performed using R package ver. 4.3.3 and Rstudio 2023.12. The ‘lavaan’ package (0.6–17) of R was mainly used.

## 3. Results

### 3.1. General Characteristics of Research Subjects

The descriptive statistics for the sociodemographic characteristics of the study subjects, adults aged 19 and older (*n* = 10,343), are shown in Table 2. To assess multicollinearity among observable variables within each latent variable group prior to conducting Structural Equation Modeling (SEM) analysis, Variance Inflation Factor (VIF) values were calculated for all variables, with all results falling below 5.31.

### 3.2. Measurement Model

The validity of the factors to be included in the structural model was first verified. For this purpose, confirmatory factor analysis (CFA) was conducted (Table 3). The outcomes of this analysis indicated a Comparative Fit Index (CFI) of 0.75 and a Tucker–Lewis Index (TLI) of 0.69, suggesting a moderate fit of the model to the data. Additionally, the Root-Mean-Square Error of Approximation (RMSEA) was found to be 0.12, coupled with a Standardized Root-Mean-Square Residual (SRMR) of 0.11. These values imply that further model refinement might be necessary to improve the fit, as the RMSEA and SRMR exceed the commonly accepted thresholds for a good fit. Construct Reliability (CR > 0.7) and Average Variance Extracted (AVE > 0.5) were derived to determine convergent validity [29]. The range of CRs was from 0.41 to 0.75 and that of AVEs was from 0.26 to 0.71.

Despite these indices, the decision to proceed with the utilization of this model is grounded in its strong theoretical underpinnings. In exploratory factor analysis, such indices would typically indicate a need for model modification. However, the current model is based on established theory, which supports the hypothesized constructs and their interrelationships. The theoretical constructs embedded within the model are well supported by the literature, providing a robust framework for interpreting the observed relationships. Consequently, while acknowledging the statistical limitations, the theoretical relevance of the model substantiates its application in this study.

### 3.3. Path Model

The deterministic factor relationships between the latent variables were verified through the structural model. The analysis results are shown in Table 4. For KM utilization, the predisposing, enabling, and need factors according to the Andersen model had significant positive effects on healthcare utilization. For WM utilization, enabling and need factors also had significant positive effects, while predisposing factors had a significant negative effect (β = −0.14, *p* < 0.01). The direction of the predisposing factor’s effect on healthcare utilization appears to be less pronounced compared to the need and enabling factors. The observed negative effect of the predisposing factor on WMHCU suggests that the predisposing factors influencing WMHCU and KMHCU may differ significantly, highlighting potential variations in demographic or contextual determinants between these two forms of healthcare utilization.

**Figure 2:**
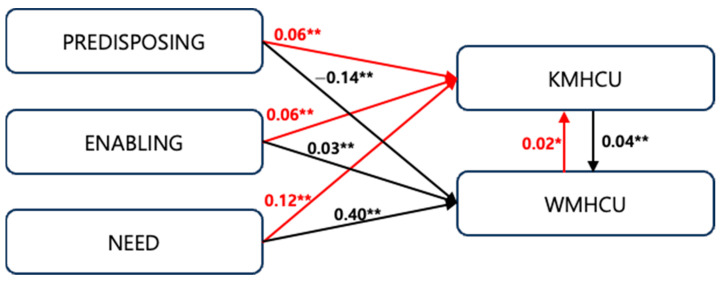
Path diagram for the determinants of healthcare utilization. (Red: paths to KMHCU, Black: paths to WMHCU). *: <0.1, **: <0.05, KMHCU: Korean medicine healthcare utilization, WMHCU: Western medicine healthcare utilization.

The path analysis of KMHCU and WMHCU showed that predisposing, enabling, and need factors independently influenced both KM and WM utilization, and that KM and WM utilization interacted to increase each other’s utilization (Figure 2). The calculated minimum detectable effect (MDE) size was 0.017 (N = 10,343, S.D. = 0.63, β = 0.05, statistical power = 0.8) and all coefficients in the model exceeded this threshold. This indicates that all estimated effects in the model are statistically detectable and robust given the sample size and statistical power, supporting the validity of the identified relationships.

### 3.4. Mediating Effect

The results of decomposing the mediating effects of exogenous variables (predisposing factors, enabling factors, need factors) on KM and WM utilization are shown in Table 5. In this analysis, the mediating effect on KMHCU involves the initial utilization of WM, which then mediates the secondary KMHCU. Similarly, the mediating effect on WMHCU entails the initial utilization of WM, which then leads to the secondary KMHCU.

First, predisposing factors did not show consistent, statistically significant indirect mediating effects on both KMHCU and WMHCU. Second, enabling factors had significant positive effects on WMHCU through KMHCU (0.002, *p* < 0.01). Lastly, need factors had significant positive indirect effects on both KM utilization (0.01, *p* < 0.1) and WM utilization (0.005, *p* < 0.01).

## 4. Discussion

The current study aimed to elucidate the determinants of healthcare utilization in South Korea’s dual healthcare system, encompassing both WM and KM, through the Andersen Model. This study conducted a path analysis using structural equation modeling, followed by mediation analysis to determine the magnitude of the effect for each pathway. The findings from this study offer several significant insights into the patterns and determinants of healthcare utilization.

Firstly, the results showed that within the same national healthcare system of South Korea, enabling factors and need factors of healthcare utilization according to the Andersen model influenced both KMHCU and WMHCU in the same direction. In the perspective of indirect effects, need factors had strong positive effects on healthcare utilization of both KM and WM, while the enabling factor did not show any significant effect on KMHCU. It is confirmed that need factors, such as chronic diseases and disability, self-rated health status, and the need for care-giving significantly affected the utilization of both WM and KM. This underscores the critical role of need factors in determining healthcare utilization, indicating that individuals utilizing not only WM but also KM are those with significant healthcare needs.

The second finding reveals the effect of the predisposing factor in the opposite direction. This suggests that the characteristics of patients who initially choose WMHCU differ fundamentally from those who prioritize KMHCU, pointing to fundamental differences in the demographic or contextual factors influencing their healthcare choices.

The third finding highlights that KMHCU and WMHCU exert mutual positive influences on each other. This contradicts the common assumption that increased utilization of one type of healthcare would reduce the utilization of its counterpart. Instead, it indicates that a higher utilization of one form of healthcare leads to greater overall healthcare utilization. This result suggests that individuals may turn to KM not solely as an alternative to WM but as a complement to address specific needs, such as pain management, well-being, and mental healthcare. This complementary use likely reflects the broader healthcare demands of individuals with multiple and diverse needs, some of which may be viewed as treatable by both systems.

This study represents the first exploratory attempt to investigate the determinants and mutual relationships of both WM and KM within the contexts of Korea’s dual healthcare system. As an initial investigation, it acknowledges several limitations and highlights the need for further research to address unresolved challenges and refine the model. The Andersen Behavioral Model of Health Services Use, employed in this study, is a representative socio-behavioral framework developed to explain healthcare utilization. Over time, the model has undergone several stages of development. The Andersen Model can be categorized into its earlier version, which focuses on predisposing, enabling, and need factors as predictors of healthcare utilization, and an expanded version that incorporates the prediction of health outcomes, such as consumer satisfaction and health status [30]. This study primarily adopts the initial Andersen Model, using individuals as the unit of analysis, to align with its exploratory nature [2]. Future research should consider extending the framework to include multilevel models that reflect contextual effects and the prediction of health outcomes as outlined in the expanded version.

Secondly, this study uses cross-sectional data from 2020 so that causality cannot be confirmed for the relationship between variables. However, the path analysis in this study presented a model-based on statistical causality, and future studies need to more clearly verify causality using longitudinal studies or data from repeated surveys.

Additionally, it should be noted that predisposing and enabling factors may exert a moderating effect on the relationship between KMHCU and WMHCU. This study attempted to explain this mediating effect by employing a non-recursive model through indirect pathways. Nevertheless, future research could investigate this potential interaction effect in greater depth by utilizing a more sophisticated modeling approach.

## 5. Conclusions

In conclusion, this study contributed through constructing and proposing a dynamic model that takes into account the mutual interaction of WM and KM by reflecting the real-world healthcare utilization patterns that use WM and KM in a complementary and alternative way. To this end, based on the Anderson model, a path model was constructed that simultaneously considered WMHCU and KMHCU, and the significance of the direct effect and indirect effect for the model was derived. The mediation analysis further highlighted the complex interplay in determining the healthcare utilization of WM and KM. The indirect effects mediated by the counterpart healthcare utilization underscore the need to consider these mutual influences in policymaking and healthcare research. 

The findings reveal that when traditional medicine functions within the national healthcare system, both Western medicine and traditional medicine are influenced by similar determinants in the same environment. Future research should explore longitudinal data to confirm these findings and investigate potential moderating effects of predisposing and enabling factors on WM and KM interactions.

## Figures and Tables

**Table 1:** Measurement variables of the Anderson Model in Western medicine and Korean medicine utilization.

Category	Western Medicine	Korean Medicine
Predisposing Factor	- Age, gender, marital status [14]	- Age and gender [15] - Gender, marital status [16]
	- Education level [17]	- Education level [17]
	- Employment status [18]	
		- Residence area (urban vs. rural) [19]
Enabling Factor	- Insurance coverage, type of medical security [14,20]	- Private Health Insurance [15]
	- Economic activities, income level [14]	
	- Accessibility to healthcare facilities [18]	-Accessibility to traditional Korean medicine facilities [16]
		Cost perception of KM treatment [14]
Need Factor	- Chronic illness, disability status [14,21]	- Presence of chronic diseases [22]
		- Severity of conditions [20]
	- Perceived health status [23]	
	- Stress levels [24]	- Stress levels [25]

**Table 2:** General characteristics of research subjects (*n* = 10,343).

Variable		Category	Mean ± Std Dev/Count (%)
PREDISPOSING	AGE		62.04 ± 16.30
	SEX	Male (ref.)	4582 (44.30%)
		Female	5762 (55.70%)
	MARRIAGE	Yes (ref.)	7390 (71.44%)
		No	2954 (28.56%)
	EDUCATION	Non (ref.)	333 (3.22%)
		Elementary school	3070 (29.68%)
		Middle school	3103 (30.00%)
		High school	1443 (13.95%)
		University	1965 (19.00%)
		Graduate school	430 (4.16%)
	REGION	Capital (ref.)	919 (8.88%)
		Metropolitan	3546 (34.28%)
		Country	5879 (56.83%)
ENABLING	PHI	No (ref.)	2761 (26.69%)
		Yes	7583 (73.31%)
	OCCUPATION	Economically inactive group (ref.)	1780 (17.21%)
		Manual	4786 (46.27%)
		Non-manual	3778 (36.52%)
NEED	CHRONIC		1.45 ± 1.56
	DISABILITY	No (ref.)	9565 (92.47%)
		Yes	779 (7.53%)
	CARE	No (ref.)	10,064 (97.29%)
		Yes	280 (2.71%)
	SRH	Excellent (ref.)	355 (3.43%)
		Good	3157 (30.52%)
		Fair	4840 (46.79%)
		Bad	1802 (17.42%)
		Very bad	190 (1.84%)
KMHCU	DAYS OF OUTPATIENT CARE		1.89 ± 8.22
	OUTPATIENT CARE COST *		31,545.96 ± 173,229.68
WMHCU	DAYS OF INPATIENT CARE		0.20 ± 0.66
	INPATIENT CARE COST *		239,944.61 ± 1,133,729.94
	DAYS OF OUTPATIENT CARE		16.81 ± 22.70
	OUTPATIENT CARE COST *		310,220.96 ± 522,824.83

* 1000 KWN, PHI: Private Health Insurance, SRH: self-rated health, KMHCU: Korean medicine healthcare utilization, WMHCU: Western medicine healthcare utilization.

**Table 3:** Confirmatory factor analysis.

			EST.STD.	SE	CR	AVE
AGE	→	Predisposing	0.84 **	0.01	0.75	0.71
SEX	→		0.11 **	0.01		
MARRIAGE	→		0.03 **	0.01		
EDUCATION	→		0.77 **	0.01		
REGION	→		0.11 **	0.01		
PHI	→	Enabling	0.54 **	0.01	0.41	0.26
OCCUPATION	→		0.50 **	0.01		
# of CHRONIC DISEASE	→	Need	0.82 **	0.01	0.70	0.57
DISABILITY	→		0.29 **	0.01		
CARE	→		0.30 **	0.01		
SRH	→		0.50 **	0.01		
DAYS of OUTPATIENT CARE	→	KMHCU	1.21 **	0.04	0.56	0.49
OUTPATIENT CARE COST	→		0.70 **	0.02		
DAYS of INPATIENT CARE	→	WMHCU	0.95 **	0.01	0.61	0.51
INPATIENT CARE COST	→		0.95 **	0.01		
DAYS of OUTPATIENT CARE	→		0.32 **	0.01		
OUTPATIENT CARE COST	→		0.23 **	0.01		

**: <0.05, PHI: Private Health Insurance, SRH: self-rated health, CR: Construct Reliability, AVE: Average Variance Extracted.

**Table 4:** Path analysis using Structural Equation Modeling.

Path		Standard Estimate	Std.Err	z-Value	P (>|z|)	CI. Lower	CI. Upper
KMHCU ~	PRED	0.06 **	0.02	2.55	0.01	0.01	0.11
	ENAB	0.06 **	0.02	3.37	<0.01	0.02	0.09
	NEED	0.12 **	0.02	5.14	<0.01	0.08	0.17
WMHCU ~	PRED	−0.14 **	0.03	−4.13	<0.01	−0.21	−0.08
	ENAB	0.03 **	0.01	2.13	0.03	0.00	0.06
	NEED	0.40 **	0.04	10.64	<0.01	0.32	0.47
KMHCU ~	WHCU	0.02 *	0.01	1.88	0.06	0.00	0.05
WMHCU ~	KHCU	0.04 **	0.01	3.80	<0.01	0.02	0.06

*: <0.1, **: <0.05, KMHCU: Korean medicine healthcare utilization, WMHCU: Western medicine healthcare utilization, PRED: predisposing factor, ENAB: enabling factor, NEED: need factor.

**Table 5:** Mediating effect of healthcare utilization.

Healthcare Utilization	Path	Std. Estimate	Std. Error	z-Value
KMHCU	PRED ~ WMHCU ~ KMHCU	−0.003 *	0.002	−1.76
	ENAB ~ WMHCU ~ KMHCU	0.001	0.001	1.41
	NEED ~ WMHCU ~ KMHCU	0.01 *	0.01	1.89
WMHCU	PRED ~ KMHCU ~ WMHCU	0.002	0.001	1.63
	ENAB ~ KMHCU ~ WMHCU	0.002 **	0.001	2.40
	NEED ~ KMHCU ~ WMHCU	0.005 **	0.001	5.47

*: <0.1, **: <0.05, KMHCU: Korean medicine healthcare utilization, WMHCU: Western medicine healthcare utilization, PRED: predisposing factor, ENAB: enabling factor, NEED: need factor.

## Data Availability

Publicly available datasets were analyzed in this study. These data can be found from the Korea Health Panel survey, which is available to the scientific community with a signed data access agreement from the Korea Institute for Health and Social Affairs and the National Health Insurance Service database (https://www.khp.re.kr:444/eng/main.do (accessed on 5 March 2024)).
